# 1-Aminocyclopropane-1-carboxylic acid deaminase producing beneficial rhizobacteria ameliorate the biomass characters of *Panicum maximum* Jacq. by mitigating drought and salt stress

**DOI:** 10.1038/s41598-018-35565-3

**Published:** 2018-11-30

**Authors:** Garima Tiwari, P. Duraivadivel, Satyawati Sharma, Hariprasad P.

**Affiliations:** 1 Biochemistry Lab, Centre for Rural Development and Technology, Indian Institute of Technology Delhi, Hauz Khas, New Delhi, 110016 India; 2 Environmental Biotechnology Lab, Centre for Rural Development and Technology, Indian Institute of Technology Delhi, Hauz Khas, New Delhi, 110016 India

## Abstract

1-Aminocyclopropane-1-carboxylic acid (ACC) is a precursor molecule of ethylene whose concentration is elevated in the plant subjected to biotic and abiotic stress. Several soil microorganisms are reported to produce ACC deaminase (ACCd) which degrades ACC thereby reducing stress ethylene in host plants. This study is aimed to apply ACCd producing beneficial rhizobacteria to improve biochemical parameters and cell wall properties of *Panicum maximum* exposed to salt and drought stress, focusing on bioethanol production. Thirty-seven ACCd producing bacteria isolated from rhizospheric soil of field grown *P. maximum* and 13 were shortlisted based on their beneficial traits (root colonization, production of indole acetic acid, siderophore, hydrogen cyanide, phosphate solubilization, biofilm formation, tolerance to salt and Polyethylene glycol) and a total score obtained. All shortlisted bacteria were found significant in enhancing the plant growth, water conservation, membrane stability, biocompatible solutes and protein, phenolic contents and photosynthetic pigments in plants grown under stress conditions. Cell wall composition (Cellulose, Hemicellulose and Lignin) of the treated plants grown under stress conditions recorded a significant improvement over their respective controls and found equivalent to the plants grown under normal circumstances. Biomass from bacterial treatment recorded higher total reducing sugars upon pre-treatment and hydrolysis, and theoretical bioethanol yield.

## Introduction

At present India represents 18% of the world human population and 15% livestock population accommodated on 2.4% of land mass. Indian population is expected reach 1.8 billion in next 30 years imposing severe pressure on agricultural productivity and production. Earlier under the similar situation, to cope up the increasing demand and attain self-sustainability, India agrarian system adopted several new initiatives. Agrochemicals played the prime role in increasing agricultural productivity, but its improper usage severely affected the soil health by reducing beneficial microbes and total organic carbon^1,2^. One of the significant concerns with Indian agriculture is the decrease in productivity and production due to the increase in the degree of land degradation and area of degraded land^3^. Similarly, climate change and altered rain pattern imposing a severe threat to plant health and productivity. Among all the abiotic stresses, drought and soil salinization are the major obstacles for plant growth and health^4–6^.

On the other hand, due to excessive consumption of petroleum products and emissions of greenhouse gases, the whole world has driven the interest in renewable energy emphasizing the bioenergy. Biomass-based energy products such as bioethanol, biogas, biodiesel, etc. are already being commercially produced and used. From economic and environmental points of view, lignocellulosic bioethanol (second generation biofuel), shows many potential advantages in comparison to starch and sugar based bioethanol (first generation biofuel). In this regard *Panicum maximum* jacq. (Family-Poaceae), a perennial grass distributed mainly in the tropics and subtropics^7^ could be a potential source of lignocellulose biomass. Due to its multi-cut nature, ease of propagation, fast growth, good yield, low input requirement and wide adaptability under different agroclimatic conditions of India, this grass could offer a sustainable and economical alternative option for bioethanol production in future.

The cell wall is the major component of lignocellulose biomass which is a potential source of energy. It mainly composed of cellulose, hemicellulose, and lignin in variable amount. Cellulose and hemicellulose polymers are strongly linked with the lignin by hydrogen and covalent bonds, which makes the whole structure very robust and recalcitrant to depolymerization^8,9^. Hence, obtaining fermentable sugars from the plant cell wall is considered a significant obstacle in the process of lignocellulosic bioethanol production. By nature, plant cell wall is meant to provide mechanical strength and protect the plant against various biotic and abiotic stresses. Plants grown under any deviation from their normal growing conditions shows respond by modifying their morphological, physiological and biochemical parameters. Early research on modulation of lignin biosynthesis under stress conditions revealed the upregulation of lignin biosynthesis enzyme and higher accumulation of lignin in cell wall under different stress^10^.

One of the futuristic approaches is the genetic modification of plants to yield better quality cell wall which serves as better feedstock for biofuel production. However, most of the earlier studies with cell wall genetic modifications lead to defects in plant growth, physiology and biochemistry thereby reducing biomass yield and survivability^11–13^. Hence it becomes essential to find out a long-lasting, eco-friendly solution to maintain the healthy plant growth and quality of biomass under stressful conditions.

At the hormonal level, ethylene plays multiple roles in the regulation of plant growth and development, and its increased biosynthesis under stress modulates the plant response which leads to restraining in biomass production^14–16^. Hence managing ethylene level in plants is a challenge under stress conditions. In nature, always plants are assisted by several beneficial microbes to withstand/tolerate/resist biotic or abiotic stress. 1-aminocyclopropane-1-carboxylic acid deaminase (ACCd) producing beneficial rhizobacteria reduces the stress ethylene level by degrading ACC (an immediate precursor of ethylene) into α-ketobutyrate and ammonium which is further used by microbes as a carbon and nitrogen source^17^. These findings opened a new avenue and showed a possible strategy for managing plant health and growth under stress conditions.

In this study, we report the enhanced plant growth and biomass characters of *Panicum maximum* by employing ACCd beneficial rhizobacterial isolates endowed with multiple advantageous traits under drought and salt stress conditions. The findings of this investigation would offer us an opportunity to exploit some potential ACCd rhizobacteria as bioinoculant to increasing the quality biomass yield of *P. maximum*, on degraded and marginal lands and further their utilization for bioethanol production.

## Results

Supplementary Tablescterization of ACCd isolates. In the presents study, 37 bacterial isolates from 25 rhizospheric soils were able to grow in DF minimal media amended with ACC as the sole nitrogen source. Quantification of ACC left over in media after degradation with test bacteria revealed that the isolate 4F1 had highest ACC utilizing ability (45.36%) followed by 11G (45.03%) > 20B (36.72%) > 18D (36.27) > 7JG (36.06) > 5C (35.03) > 5JB (33.01). The bacterial cell lysate of isolate 11G recorded the highest activity of 3072 nm/mg protein/h followed by 14P > 20B > 7C > 5JB > 11-2I > 7D > 4F1 (Table 1). *Bacillus licheniformis* and *Bacillus subtilis* are the major group of bacteria identified from the rhizosphere of *P. maximum* capable of degrading ACC (Supplementary Table S1).

**Table 1:** Quantities analysis of selected beneficial traits of ACCd rhizobacteria.

Isolates	ACC (% degradation)	ACC activity (nm/mg protein/h	IAA (µg/ml)	Biofilm (OD at 570 nm)	PS (mm)	Abiotic stress tolerance		Antagonism (% Inhibition)		Plant growth promotion			
						W (IC_50_)	S (IC_50_)	*F.v*	*A.f*	SL (cm)	RL (cm)	DW (g/seedling)	LA (mm^2^)
Control	—	—	—	—	—	—	—	—	—	16.24 ± 0.59^y^	8.49 ± 0.27°	0.28 ± 0.02^stu^	40.04 ± 0.12^g^
5JB	33.01 ± 0.93^c^	1815.68 ± 2.99^c^	53.43 ± 0.77^b^	1.96 ± 0.01	10 ± 1.01	13.082 ± 0.29^b^	62.55 ± 0.31^e^	16.66 ± 1.01	0.0	23.47 ± 0.80^j^	16.47 ± 0.12^bc^	0.68 ± 0.02^f^	46.6 ± 0.02^bc^
20B	36.72 ± 0.52^c^	1834.01 ± 1.07^c^	46.91 ± 1.09^c^	1.388 ± 0.01	12 ± 0.98	9.99 ± 0.31^de^	50.98 ± 1.6^g^	0.0	28.57 ± 1.1	25.87 ± 0.14^c^	15.87 ± 0.34^bcd^	0.72 ± 0.01^b^	49.3 ± 0.04^a^
7C	4.98 ± 0.43^st^	1823.82 ± 2.95^c^	29.30 ± 1.24^fgh^	0.384 ± 0.01	0.0	2.541 ± 0.1^mn^	33.738 ± 0.39^lm^	35 ± 0.98	34.28 ± 1.03	20.60 ± 0.85^q^	10.60 ± 0.09^mn^	0.37 ± 0.02^tuv^	41.6 ± 0.04^def^
11-2E	14.13 ± 1.1^lmn^	550.91 ± 1.36^m^	16.04 ± 1.07^p^	0.156 ± 0.02	15 ± 1.1	7.148 ± 0.32^gh^	54 ± 0.98^f^	67.33 ± 0.99	0.0	24.23 ± 1.13^h^	14.23 ± 0.73^hi^	0.28 ± 0.05^stu^	42.03 ± 0.01^de^
22F2	23.43 ± 1.5^fg^	1561.09 ± 0.74^e^	32.13 ± 1.03^e^	1.884 ± 0.006	7 ± 0.89	7.614 ± 0.45^g^	67.6 ± 0.97^d^	62.66 ± 1.02	57.14 ± 1.23	23.55 ± 0.59^i^	14.55 ± 0.33^gh^	0.29 ± 0.05^qrs^	44.23 ± 0.05^cd^
22F1	8.30 ± 1.4^r^	561.09 ± 0.93^qr^	40.82 ± 1.06^d^	0.21 ± 0.01	0.0	2.92 ± 0.08l^mn^	34.99 ± 1.7^k^	0.0	0.0	16.81 ± 0.89^y^	8.81 ± 0.14°	0.31 ± 0.03°	40.99 ± 0.02^fg^
4F1	45.36 ± 0.43^a^	1651.73 ± 1.3^e^	56.69 ± 0.78^a^	1.20 ± 0.02	8 ± 1.2	13.07 ± 0.42^b^	43.57 ± 0.41^ij^	51.66 ± 1.0	40 ± 1.04	25.89 ± 1.04^c^	15.89 ± 0.65^bcd^	0.78 ± 0.01^a^	44.76 ± 0.07^cd^
3B	13.45 ± 0.87^mn^°	387.98 ± 0.61^p^	20.17 ± 0.38^mn^	0.15 ± 0.01	11 ± 0.87	3.61 ± 0.58^jklm^	42.46 ± 1.08^j^	0.0	0.0	20.58 ± 0.51^q^	10.58 ± 0.21^mn^	0.39 ± 0.07^l^	40.02 ± 0.05^g^
11-2F	9.34 ± 0.80^q^	287.98 ± 0.85^q^	18.86 ± 1.44^mn^°	0.19 ± 0.01	0.0	18.46 ± 0.07^a^	35.18 ± 1.08^k^	0.0	0.0	18.56 ± 0.68^u^	15.56 ± 0.18^def^	0.27 ± 0.04^uv^	39.83 ± 0.04 ^h^
20N	14.79 ± 1.1^klm^	773.93 ± 3.3^ij^	31.69 ± 0.98^ef^	0.15 ± 0.008	0.0	2.92 ± 0.19l^mn^	60.2 ± 0.61^e^	0.0	0.0	25.69 ± 1.33^d^	12.69 ± 0.13^jk^	0.26 ± 0.03^w^	41.12 ± 0.03^e^
8F	15.64 ± 0.95^jkl^	632.38 ± 1.0^k^	20.82 ± 0.61^m^	0.50 ± 0.01	0.0	5.76 ± 0.36^i^	46.17 ± 0.77^hi^	0.0	0.0	18.73 ± 0.75^u^	11.73 ± 0.09^kl^	0.2.8 ± 0.05^rst^	39.3 ± 0.05^h^
11G	45.03 ± 1.3^a^	11172.1 ± 2.5^a^	38.43 ± 0.20^de^	0.62 ± 0.008	15 ± 1.1	8.7 ± 0.61^f^	51 ± 1.00^g^	0.0	34.28 ± 1.06	26.78 ± 0.27^a^	16.78 ± 0.43^b^	0.54 ± 0.05^g^	45.87 ± 0.03^c^
2L	21.03 ± 0.45^gh^	783.09 ± 0.68^ij^	19.08 ± 0.50^mn^°	0.16 ± 0.02	0.0	8.76 ± 0.30^f^	30.58 ± 0.53^n^°	23.33 ± 0.96	0.0	20.55 ± 0.65^q^	11.55 ± 0.16^kl^	0.28 ± 0.02^rst^	42.89 ± 0.02^de^
14P	28.02 ± 1.3^d^	2119.14 ± 4.5^b^	19.08 ± 0.32^e^	3.01 ± 0.007	11 ± 0.91	9.66 ± 0.11^def^	84.61 ± 0.42^b^	50 ± 1.1	28.57 ± 0.98	24.75 ± 0.18^g^	17.75 ± 0.09^a^	0.68 ± 0.01^e^	49.34 ± 0.05^a^
3E	15.96 ± 0.46^kl^	306.51 ± 1.7^q^	15.17 ± 0.82^p^	3.51 ± 0.02	18 ± 0.95	3.33 ± 0.39^klmn^	40.72 ± 1.16^j^	50 ± 1.16	0.0	21.20 ± 0.57°	10.20 ± 0.30^n^	0.30 ± 0.03^p^	43.6 ± 0.01^d^
4F11	26.69 ± 0.85^de^	1151.73 ± 1.7^gh^	38.43 ± 0.91^de^	0.60 ± 0.02	15 ± 1.05	12.136 ± 0.16^bc^	77 ± 0.26^c^	0.0	28.57 ± 1.1	23.68 ± 0.20^i^	16.68 ± 0.14^bc^	0.67 ± 0.01^e^	45.79 ± 0.04^c^
18D	36.27 ± 1.2^b^	1284.11 ± 1.9^g^	38.04 ± 0.59^de^	1.95 ± 0.01	0.0	2.87 ± 0.45^mn^	60.6 ± 0.75^e^	0.0	34.28 ± 1.08	24.67 ± 0.37^g^	14.67 ± 0.18^fgh^	0.52 ± 0.06^h^	47.12 ± 0.01^bc^
14N	18.75 ± 0.87^hij^	724.03 ± 4.4^j^	8.43 ± 1.02^r^	0.20 ± 0.01	10 ± 1.1	2.67 ± 0.09^mn^	23.66 ± 0.78^p^	0.0	0.0	23.12 ± 0.15^l^	13.12 ± 0.16^jk^	0.42 ± 0.04^j^	41.6 ± 0.02^e^
3C	14.25 ± 0.79^klm^	827.49 ± 1.5^hij^	25.60 ± 0.65^jk^	0.19 ± 0.02	20 ± 0.99	2.67 ± 0.36^mn^	34.65 ± 0.54^k^	33.33 ± 0.94	28.07 ± 1.08	23.32 ± 0.33^k^	10.32 ± 0.29^mn^	0.27 ± 0.02^uv^	41.37 ± 0.05^e^
5C	35.03 ± 0.50^bc^	1469.45 ± 1.7^f^	24.08 ± 1.12^l^	2.92 ± 0.02	0.0	6.30 ± 0.66^gh^	48.62 ± 1.15^gh^	0.0	0.0	25.75 ± 0.39^d^	15.75 ± 0.6^cde^	0.72 ± 0.02^b^	50.02 ± 0.02^a^
20F	12.44 ± 0.79°^p^	41.75 ± 1.8^u^	29.08 ± 1.24^gh^	0.21 ± 0.02	0.0	3.65 ± 0.34^jklm^	48.31 ± 0.85^gh^	0.0	0.0	17.23 ± 0.44^x^	11.23 ± 0.13^lm^	0.26 ± 0.04^w^	39.97 ± 0.07^gh^
23*	17.47 ± 1.0^ijk^	357.43 ± 4.2^pq^	11.69 ± 0.64^q^	0.16 ± 0.002	0.0	3.5 ± 0.39^klm^	12.57 ± 1.74^q^	0.0	0.0	19.56 ± 0.25^s^	13.56 ± 0.13^ij^	0.45 ± 0.01^i^	40.55 ± 0.02^g^
18G	11.33 ± 1.9^pq^	1085.94 ± 2.3^pq^	39.73 ± 0.93^d^	0.18 ± 0.002	0.0	12.1 ± 0.62^bc^	34.9 ± 0.37^k^	0.0	0.0	23.93 ± 0.91^h^	11.03 ± 0.19^lm^	0.31 ± 0.04°	41.88 ± 0.03^fg^
4F2	16.58 ± 0.45^ijk^	851.73 ± 0.83^hi^	28.65 ± 0.18^hi^	2.97 ± 0.01	0.0	10.31 ± 0.26^d^	49.68 ± 0.59^g^	0.0	0.0	26.43 ± 0.40^b^	16.43 ± 0.27^bc^	0.45 ± 0.02^hi^	45.87 ± 0.02^c^
23G	17.04 ± 1.0^ijk^	506.10 ± 3.5^n^	31.91 ± 1.03^ef^	0.42 ± 0.006	0.0	4.30 ± 0.13^jk^	60.22 ± 0.61^e^	0.0	0.0	19.04 ± 1.01^t^	11.04 ± 0.23^lm^	0.26 ± 0.01^vw^	40.23 ± 0.05^fg^
1JF	18.90 ± 1.8^hi^	764.76 ± 3.6^ij^	27.13 ± 0.93^hij^	0.18 ± 0.009	0.0	4.65 ± 0.07^j^	54.11 ± 2.10^f^	56.66 ± 1.23	57.14 ± 1.18	20.72 ± 0.24^q^	12.72 ± 0.51^jk^	0.41 ± 0.03^k^	40.99 ± 0.04^fg^
7JG	36.06 ± 0.43^b^	550.91 ± 1.5^m^	38.21 ± 0.53^de^	0.23 ± 0.02	0.0	2.65 ± 0.18^mn^	28.98 ± 1.02°	0.0	0.0	22.70 ± 0.77^m^	12.70 ± 0.24^jk^	0.33 ± 0.05^n^	41.98 ± 0.03^e^
7D	25.03 ± 0.83^d^	1711.81 ± 2.1^def^	46.91 ± 1.17^c^	1.69 ± 0.01	10 ± 0.92	11.36 ± 0.10^c^	54.94 ± 0.69^f^	68.33 ± 1.09	0.0	24.52 ± 1.03^g^	15.52 ± 0.26^def^	0.70 ± 0.03^c^	49.82 ± 0.01^a^
24F	13.06 ± 0.79^n^°^p^	194.50 ± 3.6^t^	17.78 ± 0.54^n^°^p^	1.86 ± 0.005	0.0	3.23 ± 0.17^klmn^	33.46 ± 0.60^lmn^	0.0	0.0	17.43 ± 0.23^v^	12.43 ± 0.32^efg^	0.54 ± 0.02^g^	39.98 ± 0.02^gh^
22A	6.12 ± 0.71^rs^	805.49 ± 1.9^i^	26.04 ± 1.04^ij^	0.48 ± 0.004	0.0	2.59 ± 0.08^mn^	35.15 ± 0.42^k^	0.0	0.0	20.10 ± 0.28^r^	13.10 ± 0.07^jk^	0.31 ± 0.05°	42.34 ± 0.01^d^
5JE	2.42 ± 1.1^s^	203.18 ± 2.1^s^	21.04 ± 0.04^m^	0.48 ± 0.01	0.0	2.34 ± 0.10^n^	30.31 ± 0.48°	0.0	0.0	25.6 ± 0.10^e^	15.6 ± 0.14^def^	0.46 ± 0.05^i^	43.99 ± 0.03^cd^
20H	23.25 ± 0.24^fg^	459.266 ± 2.2°	26.91 ± 1.19^hij^	0.19 ± 0.01	0.0	3.99 ± 0.18^jkl^	41.7 ± 1.31^j^	71.66 ± 1.2	71.42 ± 1.27	20.16 ± 0.39^r^	15.16 ± 0.31^efg^	0.48 ± 0.05^h^	39.75 ± 0.04^gh^
14G	8.89 ± 1.1^qr^	203.098 ± 1.8^s^	16.26 ± 1.20°^p^	0.19 ± 0.02	0.0	3.01 ± 0.30l^mn^	30.78 ± 0.25^mn^°	0.0	0.0	21.78 ± 0.74^n^	12.78 ± 0.09^jk^	0.28 ± 0.01^stu^	41.32 ± 0.03^efg^
11-2I	24.95 ± 0.84^def^	1720.032 ± 0.4^de^	19.08 ± 0.98^mno^	3.42 ± 0.003	15 ± 1.04	9.73 ± 0.44^def^	63.22 ± 1.84^e^	58.33 ± 1.03	0.0	25.25 ± 0.88^f^	15.25 ± 0.28^efg^	0.69 ± 0.01^d^	48.95 ± 0.01^b^
18F	24.01 ± 0.88^efg^	987.032 ± 2.3^h^	24.73 ± 0.86^kl^	0.20 ± 0.006	12 ± 0.93	3.3 ± 0.36^klmn^	50.46 ± 0.61^g^	0.0	0.0	21.42 ± 0.24^o^	10.42 ± 0.12^mn^	0.29 ± 0.02^rst^	45.33 ± 0.01^c^
5JD	26.96 ± 1.0^de^	1456.566 ± 1.1^f^	44.95 ± 1.15^c^	1.36 ± 0.01	11 ± 1.09	9.05 ± 0.15^ef^	89.4 ± 1.60^a^	33.33 ± 1.27	54.28 ± 1.13	24.97 ± 1.15^g^	15.97 ± 0.22^bcd^	0.36 ± 0.03^m^	49.72 ± 0.03^a^
4A*	14.36 ± 1.0^gh^	1077.099 ± 3.6^gh^	28.56 ± 0.66^hi^	0.44 ± 0.01	0.0	6.95 ± 0.34^gh^	74.29 ± 1.18^c^	0.0	57.14 ± 1.18	20.45 ± 0.94^q^	14.45 ± 0.15^gh^	0.29 ± 0.01^qr^	41.98 ± 0.03^ef^

Values followed by different superscripts in each column are significantly different (P ≤ 0.05).

*ACC: 1-aminocyclopropane carboxylic acid; IAA: Indole acetic acid; PS: Phosphate solubilization; FO: *Fusarium verticillioides*; AF: *Aspergillus flavus*; SL: Shoot length; RL: Root length; LA: Leaf area; DW: Dry weight. Control: Seedlings raised without bacterial seed treatment.

### Characterization of ACCd bacterial isolates for beneficial traits

All test isolates were found colonizing the roots of *P. maximum* and recorded positive results for IAA production which varied from 56.69 to 8.43 µg/ml with isolates 4F1 and 14 N, respectively. 83.78% of bacteria were found forming biofilm. Highest biofilm production was recorded by isolate 3E (3.515) followed by 11-2I (3.423) > 14P (3.016) > 4F2 (2.97) > 5C (2.92) > 18D (1.951) (Table 1). Sixteen bacterial isolates found to solubilize the inorganic phosphate, and none of the test bacteria was found producing HCN. Antagonism assay results showed that 13 isolates were found to be inhibitory for the growth of *A. flavus* and 14 isolates for *F. verticillioides* while the 7 had antagonistic activity against both the test fungus (Table 1).

Twenty-seven % of bacterial isolates were able to tolerate PEG concentration above 10% (−0.3 Mpa) and isolate 11-2F was tolerant to PEG concentration as high as 15% (0.45 Mpa). Similarly, 21.6% of the bacterial isolate was found tolerating salt concentration of 50 g/L, isolates 14P and 5JD were found to endure up to 90 g/L salt concentration (Table 1).

Under normal conditions, all the test bacteria recorded various level of plant growth promotion in comparison with control. Except isolate 22F1all were able to increase the root length (RL) and isolate 14P recorded significantly (P ≤ 0.05) highest RL (17.75 cm) followed by 11G (16.78 cm). Concerning shoot length (SL) best results were recorded by isolate 11G (26.78 cm) followed by 4F2 (26.43 cm). Whereas in case of dry weight (DW) yield, 4F1 showed highest (0.78 g/seedling) which was closely followed by 5C (0.72 g/seedling) over other isolates and control (0.2 g/seedling). Similarly, leaf area (LA) was found increased significantly (P ≤ 0.05) in seedlings raised from 5C isolate treatment (50.02 mm^2^) closely followed by isolate 7D (49.82 mm^2^) > 5JD (49.72 mm^2^) > 14P (49.34 mm^2^) > 20B (49.3 mm^2^) > 11-2I (48.95 mm^2^) > 18D (47.12 mm^2^) over control (40.6 mm^2^) (Table 1, Supplementary Fig. S1).

**Figure 1:**
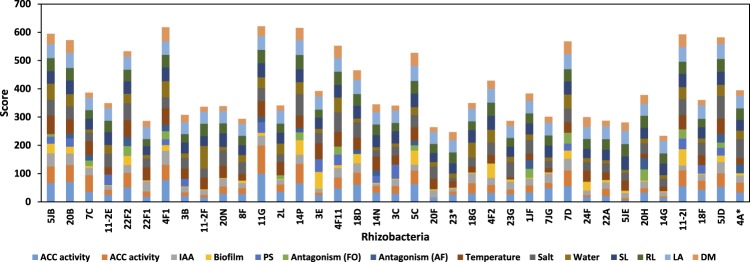
Score chart representing total scores achieved by each ACCd rhizobacterial isolates.

In our studies to find out the better bacterial strains for further screening, we developed a score chart by giving weight for each beneficial traits of bacteria (Table 2). Highest weight of 100% was given to ACCd activity, as this character was our point of interest, which ensures the selection of better ACCd producing bacteria. Other traits were given weight depending upon their importance in the present study (Table 2). Based on the weight, a score was assigned to each bacteria for each beneficial traits. The highest total score of 621.3 was recorded to isolate 11G followed by 14P (615.7) (Fig. 1). Thirteen bacteria which scored more than 400 were shortlisted for second stage screening.

**Table 2:** Weight and maximum value obtained for each beneficial traits tested for ACCd rhizobacteria.

S. No	Traits		Weight (%)	Maximum value obtained
1	ACC deaminase	Percent degradation (%)	100	45.3
		Deaminase activity (nm/mg protein/h)	100	3072
2	Root colonization (%)		100	100
3	Growth under stress	Salt stress: NaCl (g/L, IC_50_)	80	89.4
		Water stress: PEG (%, IC_50_)	80	18.46
4	Biofilm (OD at 570 nm)		60	3.51
5	Plant growth promotion under normal condition	Shoot Length (cm)	50	26.43
		Root Length (cm)	50	17.75
		Dry Weight (g/seedlings)	50	0.78
		Lear Area (mm^2^)	50	49.72
6	Indole acetic acid (µg/ml)		50	56.69
7	Phosphate solubilization (mm)		50	20.0
8	Antagonism	*A. flavus* (%)	40	71.42
		*F. verticilloides* (%)	40	71.66

### ACCd rhizobacteria induce systemic tolerance against drought and salt stress

In the second stage screening, we used several morphological, physiological and biochemical parameters of the host plant to evaluate the stress reduction capabilities of rhizobacteria.

### Plant growth parameters

**Figure 2:**
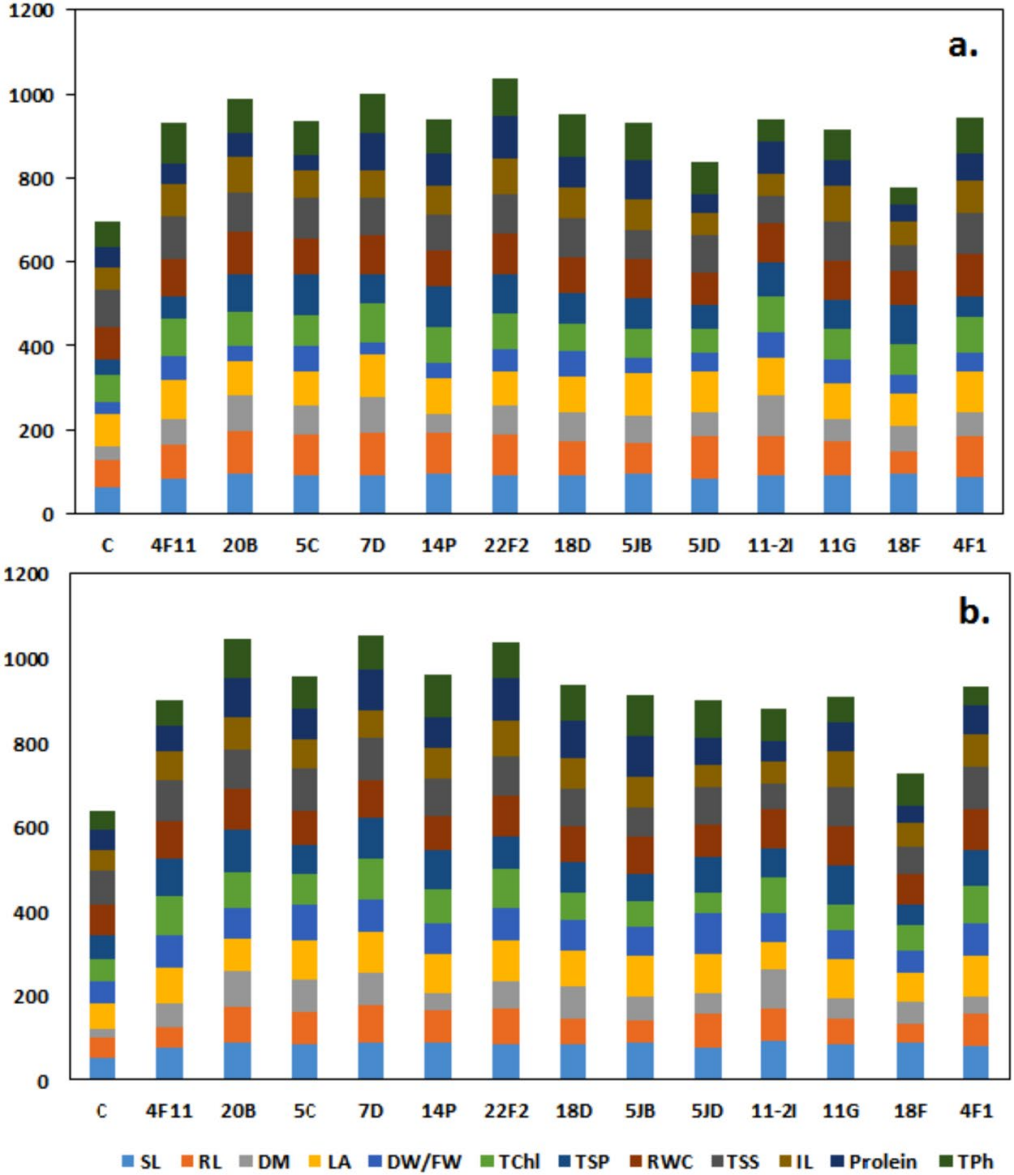
Total score achieved by selected 13 ACCd rhizobacterial isolates in improving the health and growth of host plants under drought (**a**) salt and (**b**) stress condition.

Improved growth parameters of *P. maximum* treated with selected bacteria under drought stress and salt stress represented in Fig. 2, Supplementary Fig. S2, Tables 3 and 4. Under drought conditions, isolate 20B and 7D recorded significantly (P ≤ 0.05) higher SL and RL of 18.31 cm and 10.36 cm, respectively over control and other treatments. Isolate 5JD and 7D recorded a maximum leaf area of 46.96 mm^2^ followed by 5JB > 4F1 > 4F11 > 11-2I > 14P > 11G > 20B > 18D > 18F > 22F2 > 5C. Isolate 11-2I treated seedlings recorded significantly (P ≤ 0.05) highest DM of 1.4 g/seedling while the 7D and 20B showed at par (1.28 g/seedling), in comparison with control (0.47 g/seedling). Similarly selected bacteria recorded an enhancement in growth parameters of *P. maximum* under salt stress. Significantly (P ≤ 0.05) highest of 16.7 cm SL (20B), 8.56 cm RL (7D), 11.9 g DW (11-2I) and 54.1 mm^2^ LA (4F11) were recorded with various bacterial treatments which were higher than control and other bacterial treatment.

**Table 3:** Effect of ACCd rhizobacteria treatment on morphological, physiological and biochemical characters of *Panicum maximum* grown under drought stress.

Isolates	SL (cm)	RL (cm)	DM (g seedling^−1^)	LA (mm^2^)	DW/FW*	RWC (%)	IL (%)	TSP (mg/g FW)	Proline (mg/g FW)	TSS (mg/g FW)	CP (%)	TPC (mg/g FW)	TC (mg/g FW)
N	18.91 ± 0.21^a^	9.56 ± 0.30^a^	1.48 ± 0.013^a^	46.98 ± 0.44^a^	0.09 ± 0.015^f^	85.47 ± 0.601^a^	17.09 ± 0.02^g^	3.46 ± 0.039^j^	0.069 ± 0.001^g^	79.8 ± 0.48^f^	11.2 ± 0.51^a^	3.675 ± 0.025^j^	40.87 ± 0.40^a^
C	11.61 ± 0.11^o^	6.52 ± 0.34^n^	0.47 ± 0.023^h^	35.6 ± 0.16^g^	0.30 ± 0.035^a^	64.39 ± 0.45^f^	32.98 ± 0.02^a^	5.07 ± 0.053^h^	0.102 ± 0.003^j^	112.1 ± 0.11^i^	6.02 ± 0.061^l^	4.372 ± 0.04^h^	27.01 ± 1.50^gh^
4F11	16.05 ± 0.44^n^	7.59 ± 0.44^l^	0.91 ± 0.021^e^	44.12 ± 0.40^b^	0.16 ± 0.045^g^	77.25 ± 0.41^c^	22.04 ± 0.019^e^	8.66 ± 0.09^c^	0.098 ± 0.002^gh^	127 ± 0.68^a^	8.21 ± 0.045^f^	7.004 ± 0.09^b^	36.15 ± 0.36^c^
20B	18.31 ± 0.11^b^	9.8 ± 0.41^c^	1.28 ± 0.019^c^	38.6 ± 0.31^e^	0.25 ± 0.031^b^	83.87 ± 0.19^a^	19.87 ± 0.03^f^	9.51 ± 0.06^a^	0.117 ± 0.003^f^	119.9 ± 0.78^c^	10.21 ± 0.025^c^	5.71 ± 0.01^f^	33.51 ± 0.20^cd^
5C	17.03 ± 0.66^k^	9.41 ± 0.32^d^	1.04 ± 0.018^d^	36.97 ± 0.45^f^	0.14 ± 0.06^h^	73.09 ± 0.39^d^	25.66 ± 0.04^c^	6.52 ± 0.08^g^	0.075 ± 0.004^k^	120.5 ± 0.27^c^	8.69 ± 0.01^e^	5.97 ± 0.05^e^	29.14 ± 0.53^d^
7D	17.69 ± 0.41^f^	10.36 ± 0.19^e^	1.28 ± 0.020^c^	46.97 ± 0.057^a^	0.30 ± 0.02^a^	78.09 ± 0.14^c^	26.71 ± 0.02^c^	9.55 ± 0.11^a^	0.178 ± 0.002^c^	113.9 ± 0.69^e^	10.7 ± 0.060^b^	7.06 ± 0.05^b^	38.65 ± 0.35^b^
14P	17.72 ± 0.23^e^	9.52 ± 0.27^b^	0.66 ± 0.028^g^	40.6 ± 0.47^d^	0.25 ± 0.031^b^	71.98 ± 0.54^d^	24.19 ± 0.012^d^	8.91 ± 0.08^b^	0.151 ± 0.002^d^	105.4 ± 0.73^g^	9.52 ± 0.05^d^	6.05 ± 0.02^d^	33.81 ± 0.53^cd^
22F2	17.13 ± 0.19^i^	10.02 ± 0.28^f^	1.02 ± 0.03^d^	37.99 ± 0.33^e^	0.16 ± 0.015^g^	85.6 ± 0.73^a^	20.09 ± 0.024^e^	7.05 ± 0.06^f^	0.200 ± 0.001^a^	117.9 ± 0.33^d^	11.06 ± 0.032^a^	6.511 ± 0.02^c^	34.16 ± 0.79^cd^
18D	17.45 ± 0.17^g^	7.7 ± 0.48^k^	1.04 ± 0.023^d^	38.6 ± 0.18^e^	0.14 ± 0.025^h^	73.55 ± 0.24^d^	23.65 ± 0.03^d^	6.95 ± 0.10^f^	0.147 ± .003^d^	117.2 ± 0.38^d^	7.99 ± 0.020^g^	7.304 ± 0.05^a^	26.08 ± 0.67^h^
5JB	17.75 ± 0.12^d^	7.11 ± 0.18^m^	0.99 ± 0.022^de^	46.3 ± 0.26^a^	0.23 ± 0.022^c^	81.12 ± 0.24^b^	24.33 ± 0.034^d^	5.09 ± 0.11^h^	0.186 ± .003^b^	87 ± 0.73^h^	10.63 ± 0.05^b^	6.51 ± 0.04^c^	27.68 ± 0.87^gh^
5JD	16.13 ± 0.17^m^	9.36 ± 0.12^g^	0.85 ± 0.098^ef^	46.96 ± 0.25^a^	0.2 ± 0.024^c^	67.06 ± 0.089^e^	32.89 ± 0.032^a^	7.96 ± 0.02^e^	0.093 ± .006^hi^	114.2 ± 0.97^e^	7.68 ± 0.015^h^	5.52 ± 0.03^g^	23.22 ± 1.72^i^
11-2I	17.28 ± 0.29^h^	9.05 ± 0.098^i^	1.40 ± 0.099^b^	42.33 ± 0.23^c^	0.14 ± 0.02^h^	79.12 ± 0.12^c^	32.98 ± 0.04^a^	6.7 ± 0.02^g^	0.154 ± .005^d^	80.7 ± 0.79^i^	7.03 ± 0.032^j^	4.089 ± 0.05^i^	34.76 ± 0.50^cd^
11G	17.04 ± 0.25^j^	7.84 ± 0.38^j^	0.81 ± 0.020^f^	39.89 ± 0.45^d^	0.09 ± 0.03^f^	81.99 ± 0.14^b^	20.38 ± 0.07^e^	9.01 ± 0.02^b^	0.119 ± .005^f^	117.9 ± 0.50^d^	7.27 ± 0.03^i^	5.53 ± 0.07^g^	28.83 ± 0.37^fg^
18F	18.01 ± 0.26^c^	5.07 ± 0.34^o^	0.90 ± 0.023^ef^	37.21 ± 0.30^f^	0.21 ± 0.023^d^	67.24 ± 0.64^e^	30.14 ± 0.041^b^	4.5 ± 0.024^i^	0.085 ± .004^i^	76 ± 0.16^j^	6.52 ± 004^k^	2.94 ± 0.05^k^	30.14 ± 0.35^ef^
4F1	16.8 ± 0.51^l^	9.11 ± 0.15^h^	0.82 ± 0.021^f^	46.02 ± 0.17^a^	0.2 ± 0.02^e^	85.39 ± 0.05^a^	21.87 ± 0.032^e^	8.43 ± 0.04^d^	0.127 ± .001^e^	125.8 ± 0.69^b^	8.68 ± 0.04^e^	6.05 ± 0.04^d^	36.21 ± 0.41^c^

Values followed by different superscripts in each column are significantly different (P ≤ 0.05).

SL: Shoot length; RL: Root length; DM: Dry matter; LA: Leaf area; DW/FW: Dry weight/Fresh weight; RWC: Relative water content; IL: Ionic leakage; TSP: Total soluble protein; TSS: Total soluble sugar; CP: Crude protein; TPC: Total phenol content; TC: Total chlorophyll; N: Seedlings grown under normal conditions (without bacterial treatment and stress); C: Seedlings raised under stress (without bacterial treatment).

**Table 4:** Effect of ACCd rhizobacteria treatment on morphological, physiological and biochemical characters of *Panicum maximum* grown under salt stress.

Isolates	SL (cm)	RL (cm)	DM (g seedling^−1^)	LA (mm^2^)	DW/FW*	RWC (%)	IL (%)	TSP (mg/g FW)	Proline (mg/g FW)	TSS (mg/g FW)	CP (%)	TP (mg/g FW)	TC (mg/gW)
N	18.91 ± 0.21^a^	9.56 ± 0.30^a^	1.48 ± 0.013^a^	46.98 ± 0.44^d^	0.09 ± 0.015^bcd^	85.47 ± 0.601^a^	17.09 ± 0.02i	3.46 ± 0.039^j^	0.069 ± 0.001^j^	79.8 ± 0.48^i^	11.2 ± 0.51^a^	3.675 ± 0.025^k^	40.87 ± 0.40^a^
C	9.13 ± 0.24^h^	4.55 ± 0.28^g^	0.29 ± 0.023^k^	30.2 ± 0.16^i^	0.15 ± 0.018^a^	62.55 ± 1.38^g^	34.07 ± 0.21^a^	5.20 ± 0.044^hi^	0.118 ± 0.002^h^	100.7 ± 0.30^g^	2.02 ± 0.04^m^	5.112 ± 0.001^i^	16.76 ± 1.14^gh^
4F11	13.15 ± 0.25^f^	4.6 ± 0.23^g^	0.71 ± 0.021^ef^	43.12 ± 0.34^e^	0.10 ± 0.021^bc^	75.05 ± 0.19^d^	24.14 ± 0.30^f^	8.90 ± 0.035^f^	0.140 ± 0.001^c^	120.1 ± 0.28^c^	4.2 ± 0.01^i^	8.204 ± 0.002^c^	27.84 ± 0.58^bc^
20B	16.71 ± 0.14^b^	6.58 ± 0.39^d^	1.07 ± 0.019^b^	40.58 ± 0.03^f^	0.11 ± 0.034^b^	81.39 ± 1.13^ab^	21.66 ± 0.29^g^	9.80 ± 0.029^d^	0.128 ± 0.002^e^	116.06 ± 0.25^d^	7.23 ± 0.03^b^	6.886 ± 0.001^g^	26.94 ± 0.24^bc^
5C	15.40 ± 0.17^cd^	7.48 ± 0.46^c^	0.96 ± 0.018^d^	47.19 ± 0.08^c^	0.09 ± 0.036^bcd^	70.11 ± 0.80^e^	25.66 ± 0.10^e^	6.71 ± 0.045^g^	0.119 ± 0.001^gh^	124.13 ± 0.55^a^	4.6 ± 0.015^g^	7.079 ± 0.002^f^	22.22 ± 0.29^e^
7D	15.70 ± 0.43^c^	8.56 ± 0.27^b^	0.99 ± 0.020^c^	49.24 ± 0.06^a^	0.10 ± 0.05^bc^	76.14 ± 0.87^cd^	26.51 ± 0.32^d^	9.78 ± 0.058^a^	0.196 ± 0.003^a^	122.29 ± 0.37^b^	6.07 ± 0.045^d^	8.805 ± 0.002^a^	29.77 ± 0.60^b^
14P	15.79 ± 0.38^c^	7.71 ± 0.53^c^	0.48 ± 0.028^j^	47.84 ± 0.03^bc^	0.11 ± 0.03^b^	69.08 ± 0.99^ef^	24.19 ± 0.13^f^	9.11 ± 0.017^b^	0.180 ± 0.0002^bc^	112.64 ± 0.51^e^	5.51 ± 0.055^e^	7.705 ± 0.001^e^	24.96 ± 0.59^d^
22F2	15.23 ± 0.27^cd^	8.42 ± 0.63^bc^	0.82 ± 0.03^e^	49.29 ± 0.04^a^	0.10 ± 0.04^bc^	82.11 ± 1.12^a^	20.09 ± 0.65^h^	7.45 ± 0.094^b^	0.206 ± 0.004^a^	115.58 ± 0.30^d^	6.12 ± 0.06^d^	7.647 ± 0.001^e^	28.19 ± 0.12^bc^
18D	12.41 ± 0.48^g^	5.9 ± 0.29^e^	0.96 ± 0.023^d^	43.5 ± 0.20^e^	0.10 ± 0.03^bc^	71.05 ± 0.39^e^	23.61 ± 0.15^f^	7.05 ± 0.057^gg^	0.187 ± 0.003^b^	113.62 ± 0.61^e^	4.78 ± 0.025^f^	8.611 ± 0.002^b^	19.64 ± 0.22^f^
5JB	15.95 ± 0.25^c^	5.19 ± 0.34^ef^	0.71 ± 0.022^ef^	49.11 ± 0.02^a^	0.11 ± 0.03^b^	78.31 ± 1.10^bc^	24.33 ± 0.32^f^	6.01 ± 0.046^e^	0.207 ± 0.003^a^	85.8 ± 0.29^h^	6.53 ± 0.05^c^	7.879 ± 0.001^d^	19.27 ± 0.39^f^
5JD	13.23 ± 0.40^f^	7.4 ± 0.42^c^	0.64 ± 0.098^h^	46.14 ± 0.15^de^	0.08 ± 0.01^cd^	65.15 ± 0.34^f^	32.89 ± 0.078^b^	8.07 ± 0.079^h^	0.120 ± 0.0002^g^	110.32 ± 0.22^f^	4.48 ± 0.04^g^	6.729 ± 0.007^g^	15.32 ± 1.04^h^
11-2I	13.51 ± 0.12^ef^	7.15 ± 0.44^cd^	1.19 ± 0.099^b^	32.05 ± 0.15^h^	0.10 ± 0.05^bc^	76.24 ± 1.03^cd^	32.98 ± 0.01^b^	6.91 ± 0.044^c^	0.189 ± 0.0002^b^	78.7 ± 0.60^j^	3.02 ± 0.015^k^	5.28 ± 0.006^i^	25.64 ± 0.54^cd^
11G	14.24 ± 0.16^e^	5.81 ± 0.28^e^	0.63 ± 0.020^h^	46.09 ± 0.05^de^	0.11 ± 0.03^b^	79.05 ± 0.49^bc^	20.38 ± 0.16^h^	9.21 ± 0.050^c^	0.123 ± 0.003^f^	115.87 ± 0.33^d^	3.28 ± 0.015^j^	6.914 ± 0.007^fg^	18.40 ± 0.52^g^
18F	15.11 ± 0.23^d^	3.17 ± 0.29^h^	0.68 ± 0.023^g^	34.55 ± 0.08^g^	0.15 ± 0.03^a^	65.21 ± 0.91^f^	30.14 ± 0.72^c^	4.71 ± 0.025^e^	0.089 ± 0.0006^i^	76.7 ± 0.19^k^	2.53 ± 0.011^l^	4.048 ± 0.001^j^	18.68 ± 0.27^f^
4F1	13.7 ± 0.35^ef^	7.11 ± 0.26^cd^	0.52 ± 0.021^i^	48.21 ± 0.12^b^	0.10 ± 0.03^bc^	82.3 ± 0.62^a^	21.87 ± 0.57^g^	8.67 ± 0.046^d^	0.138 ± 0.002^d^	122.81 ± 0.65^b^	5.56 ± 0.06^e^	6.428 ± 0.001^h^	27.42 ± 0.25^bc^

Values followed by different superscripts in each column are significantly different (P ≤ 0.05).

SL: Shoot length; RL: Root length; DM: Dry matter; LA: Leaf area; DW/FW: Dry weight/Fresh weight; RWC: Relative water content; IL: Ionic leakage; TSP: Total soluble protein; TSS: Total soluble sugar; CP: Crude protein; TPC: Total phenol content; TC: Total chlorophyll; N: Seedlings grown under normal conditions (without bacterial treatment and stress); C: Seedlings raised under stress (without bacterial treatment).

### Plant water content

Under drought conditions, control plants were found to lose water quicker than the bacteria treated plants. Isolate 11G found to conserve the plant water content significantly (P ≤ 0.05) (DW/FW (Dry weight/Fresh Weight) ratio: 0.09 and relative water content (RWC): 81.99%) in comparison with control drought stressed plants (DW/FW ratio: 0.30, RWC: 64.39%) (Table 3). Plants grown under normal conditions without bacterial treatment and without induced stress recorded DW/FW ratio of 0.09 and RWC of 85.47%. Under induced salt stress, isolate 18F (DW/FW ratio :0.15, RWC:65.21%) was least effective which was found equivalent to control (DW/FW ratio :0.15, RWC:62.55%) (Table 4).

### Electrolyte leakage

Exposing the seedlings to drought and salt stress lead to an excess release of electrolyte from leaf tissue (32.98% and 34.07%, respectively) when compared to seedlings grown under normal conditions (17.09%). Generally, upon bacterial treatment, a significant (P ≤ 0.05) decrease in electrolyte leakage was recorded under stress conditions. Isolate 22F2 and 20B were found significant (P ≤ 0.05) in decreasing the membrane damage in plants grown under drought and salt stress conditions, respectively (Tables 3, and 4).

### Photosynthetic pigments

Under drought and salt stress conditions the control plants recorded a significant (P ≤ 0.05) decrease in total chlorophyll content (27.01 mg/g FW and 16.76 mg/g FW, respectively) in comparison with the plant grown under normal conditions (40.87 mg/g FW, respectively). Plants treated with bacteria recorded various level of chlorophyll content in between control and normal plants. Among all bacterial treatments, isolate 7D (38.65 mg/gm FW) showed best results in maintaining chlorophyll content under drought stress followed by 4F1 > 4F11 > 11-2I > 22F2. Similarly, under drought stress isolate 7D recorded a highest of 29.77 mg/gm FW chlorophyll followed by 22F2 > 4F11 > 4F1 > 20B (Tables 3, and 4).

### Proline, total soluble sugar and total soluble protein

Proline, total soluble sugar (TSS) and total soluble protein (TSP) content considerably increased in plants treated with bacteria exposed to stress conditions in comparison to plants grown under normal and control conditions. Under drought stress, significant increase (P ≤ 0.05) in proline (0.20 mg/g FW), TSS (113.9 mg/g FW) and TSP (9.51 mg/g FW) was recorded with isolates 22F2, 4F11 and 20B, respectively (Table 3). Similarly, under salt stress, significant increase (P ≤ 0.05) in proline (0.207 mg/g FW), TSS (124.0 mg/g FW) and TSP (9.78 mg/g FW) was recorded with isolates 5JD, 5C and 7D, respectively (Table 4).

### Total phenol determination

In comparison with plants grown under normal conditions, total phenol was found higher in all bacterial treatments and control. Plant treated with isolate 18D recorded significantly (P ≤ 0.05) higher level of 7.304 mg/g FW when exposed to drought stress. However, under salt stress highest of 8.805 mg/g FW was recorded with isolate 7D. Plants grown under normal conditions without any induced stress recorded a least of 3.67 mg/g FW (Tables 3 and 4).

In our studies to choose better rhizobacteria, results of each parameter mentioned earlier were compared with the plants grown under normal conditions by giving a weight of 100% to each character. A bacteria scoring close to 1200 were selected for further studies (Fig. 2). The score for each treatment was calculated as shown in Fig. 2a,b. Under both drought and salt stress isolate 7D recorded highest of 1007.1 and 1052.3, respectively.

### Cell wall composition

**Figure 3:**
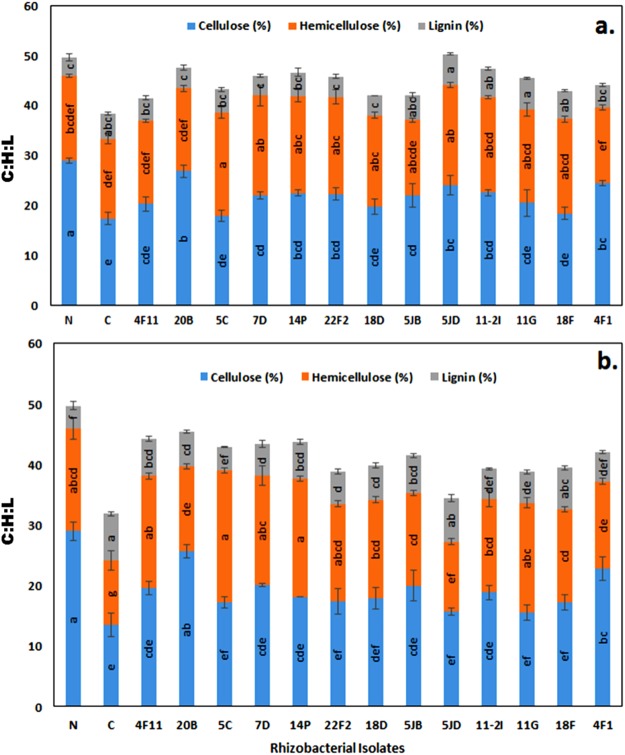
Variation in cell wall composition of *P. maximum* receiving various treatments grown under drought (**a**) and salt (**b**) stress condition.

As expected plants grown under stress conditions (drought and salt) recorded decreased cellulose content and increased lignin content in their cell wall. Under normal conditions, plant cell wall showed 29.01% cellulose, 16.96% hemicellulose and 3.73% lignin. However, when these plants were exposed to drought stress, the cellulose and hemicellulose content was reduced to 17.42% and 15.91% respectively, and lignin content was increased to 5.03% (Fig. 3a). Similarly, under salt stress the cellulose and hemicellulose content was reduced to 13.5% and 10.65%, respectively and lignin content was increased to 7.68% (Fig. 3b). Plants treated with bacteria recorded various level of improvement in cell wall composition which was indicated by increased cellulose and hemicellulose and decreased lignin content in comparison with control plants, under both drought and salt stress conditions. Among the 13 bacteria used, isolate, 4F1, 7D, 22F2 and 20B recorded significant improvement in cell wall composition under both stress conditions.

### Ethanol yield

Upon pretreatment and enzymatic hydrolysis plant grown under normal conditions recorded a highest of 359.8 μg/g of total reducing and theoretical ethanol yield of 23.29 ml/100 g dry biomass (conversion percentage, 78.25). Among the bacteria treated seedling, under drought stress isolate 20B recorded the highest total reducing sugar of 334.8 μg/g and ethanol yield of 21.68 ml/100 g biomass (conversion percentage, 77.0). Whereas, under salt stress isolate 22F2 recorded highest of 304.8 μg/g total reducing sugar and ethanol yield of 19.7 ml/100 g dry biomass (conversion percentage, 77.20). Control plants recorded the lowest total reducing sugar and bioethanol under both drought and salt stress conditions (Table 5).

**Table 5:** Potential of *Panicum maximum* biomass receiving various treatments for bioethanol production.

Treatments	Drought				Salt			
	Cellulose and Hemicellulose (μg/g)^$^	Total Reducing Sugar (μg/g)^@^	Ethanol Yield* (ml/100 g dry biomass)	% Conversion	Cellulose and Hemicellulose (μg/g)^$^	Total Reducing sugar (μg/g)^@^	Ethanol Yield* (ml/100 g dry biomass)	% Conversion
N	459.84 ± 4.1^a^	359.84 ± 5.1^a^	23.29737	78.25331	459.84 ± 32.7^a^	359.84 ± 18.1^a^	23.29737	78.25331
C	333.3 ± 21.4^e^	233.3 ± 7.5^f^	15.10471	69.997	241.56 ± 3.8^f^	141.56 ± 14.1^h^	9.165118	58.60242
4F11	370.44 ± 16.9d^e^	270.44 ± 11.9^e^	17.50929	73.00508	381.24 ± 11.9^bcd^	281.24 ± 16.5^bc^	18.20852	73.7698
20B	434.88 ± 19.0^abc^	334.88 ± 11.1^ab^	21.68137	77.00515	397.32 ± 11.0^b^	297.32 ± 8.6^b^	19.2496	74.83137
5C	386.4 ± 20.0^bcde^	286.4 ± 10.5^de^	18.5426	74.12008	390.36 ± 13.9^bc^	285.36 ± 18.1^bc^	18.47526	73.10175
7D	420.44 ± 25.5^abcd^	322.44 ± 6.4^bc^	20.87596	76.69109	381.36 ± 18.1^bcd^	291.36 ± 5.2^b^	18.86372	76.40025
14P	419.76 ± 18.8^abcd^	319.76 ± 15.9^bc^	20.70245	76.17686	376.44 ± 3.5^bcd^	276.44 ± 5.8^bcd^	17.89775	73.43534
22F2	417.6 ± 9.8^abcd^	327.6 ± 2.7^b^	21.21004	78.44828	394.8 ± 17.8^b^	304.8 ± 1.2^b^	19.73388	77.20365
18D	380.88 ± 16.8^def^	280.88 ± 7.1^e^	18.18521	73.74501	341.88 ± 22.6^bcd^	241.88 ± 16.2^def^	15.66021	70.74997
5JB	371.52 ± 23.4^de^	271.52 ± 9.1^e^	17.57921	73.08355	352.92 ± 29.2^bcd^	252.92 ± 8.3^cdef^	16.37498	71.66497
5JD	440.88 ± 20.3^ab^	290.88 ± 3.5^cde^	18.83265	65.97714	272.64 ± 10.2^e^	172.64 ± 8.5^g^	11.17735	63.3216
11-2I	416.88 ± 6.3^abcd^	316.88 ± 8.0^bcd^	20.51598	76.01228	343.2 ± 8.5^bcd^	243.2 ± 3.1^def^	15.74567	70.86247
11G	392.4 ± 12.8^bcd^	292.4 ± 3.1^cde^	18.93106	74.5158	336.84 ± 6.4^cd^	236.84 ± 6.2^ef^	15.3339	70.31231
18F	373.08 ± 7.1^de^	273.08 ± 13.1^e^	17.68021	73.1961	325.8 ± 9.8^d^	225.8 ± 9.0^f^	14.61913	69.30632
4F1	396.48 ± 4.2^bcd^	296.48 ± 15.9^cde^	19.19521	74.77805	371.88 ± 21.3^bcd^	271.88 ± 7.6^bcde^	17.60252	73.10961

Values followed by different superscripts in each column are significantly different (P ≤ 0.05).

^@^Total Reducing sugar (μg/g) was estimated after 2% NaOH, 121 °C, and 1 h pre-treatment and enzymatic hydrolysis.

^$^Cellulose and hemicellulose (μg/g) content of plants before pretreatment.

*Theoretical ethanol yield (ml/100 g) was calculated from total reducing sugars after enzymatic hydrolysis, assuming that the theoretical ethanol yield for fermenting is

0.511 g per g of hexose or pentose and by multiplying with specific volume of ethanol i.e. 1.267 ml per g (Vogel *et al*.^56^).

## Discussion

Employment of beneficial rhizobacteria for the improvement of plant health and growth and to improve the quality and quantity of food crop was frequently reported. This approach is acknowledged as one of the futuristic strategies which sustainably feed the increasing global population. On the other hand, fuel is as much as equivalent to food in our daily life. At present among the biofuels, use of lignocellulosic biomass is gaining importance as it is abundantly produced across the globe and is not compete with the food. Most of the early research works were carried out in the area of processing and conversion of lignocellulosic biomass to biofuel. However, research about the enhancing the biomass characters sustainably, especially by employing the beneficial rhizobacteria for biofuel production are sparingly reported. The beneficial rhizobacteria endowed with multiple traits would indeed facilitate the production of higher quality biomass for biofuel applications in a more sustainable way especially on degraded and marginal lands by alleviating biotic and abiotic stress^18,19^.

In past several bulk soil/rhizosphere bacteria were reported to have ACCd activity and their interaction with host plant lead to the improvement in growth and health under various biotic and abiotic stress^20,21^. Despite having ample knowledge and bacterial isolates with ACCd activity, its field applicability and availability at the commercial level is posing a hindrance in expanding this research into grass root level.

Biological management of biotic and abiotic stress is a very delicate process which depends on several factors such as, the source of biocontrol agents, beneficial traits, host plant, it’s adaptability and functioning at the particular environment, etc. Hence more emphasize should be given to screening procedure and selection of best biocontrol agents with the desired traits^22–25^. Also, native biocontrol agents are preferable than exotics because of their better adaptability.

In the present study, we isolated a total of 37 bacteria which showed the ability to utilize ACC as nitrogen source from the rhizosphere of *P. maximum* and evaluated for their potential to alleviate abiotic stress and enhance biomass characters for biofuel applications. Bacterial produced ACCd at the close vicinity of the root is known to reduce the ethylene concentration by catalyzing the degradation of ACC into α-ketobutyrate and ammonia^26–28^. Also, bacterial ability to produce ACCd and its catalyzing rate varies from isolate to isolate under *in vitro* conditions. But under *in vivo* conditions, it is not only ACCd which is acting on the plant, along with it other beneficial traits individually or in combinations are involved in improving plant health and growth under stress^29^. Additionally, their ability to survive and grow under various salt and drought conditions make them suitable to use in degraded and marginal lands.

In our studies, along with ACC utilization, the bacteria found to have several beneficial traits such as plant growth promotion, IAA production, biofilm formation, phosphate solubilization, siderophore production and antagonistic activity against plant pathogenic fungus. Advantages of multi-trait beneficial rhizobacteria in improving the plant biomass and yield suppressing/tolerating biotic and abiotic stress were reported earlier in several plants systems^30–32^. In our studies to find out the better bacterial strains for further screening, we developed a score chart by giving weight for each beneficial trait of bacteria (Table 2). Highest weight of 100% was given to ACCd activity, as this trait was our point of interest which ensures the selection of better ACCd producing bacteria. Other traits were given weight depending upon their importance in the present study. Based on the obtained score chart 13 bacteria which scored more than 400 were shortlisted for second stage screening.

In the second stage screening, we used several morphological, physiological and biochemical parameters of the host plant to evaluate the stress reduction capabilities of rhizobacteria. Plants exposed to drought and salt stress make an effort to survive by modifying morphological physiological and biochemical characters (increased root length, decreased shoot length, decreased leaf area and increased thickness, reduced stomatal number, Relative water content, increased accumulation compatible solutes, increased antioxidant enzymes, elevated abscisic acid biosynthesis, etc.). The degree of these modifications determines the survivability and growth of plants^33,34^.

Earlier researchers used several such traits as indicators to measure stress tolerance in plants. In our studies to choose better rhizobacteria, values of characters as mentioned earlier were compared with the plants grown under normal conditions by giving the weight of 100% to each character. As totally 12 characters were considered, a bacterium which scores close to 1200 is considered as best for further studies (Fig. 2).

*Panicum maximum* cultivars were analyzed for their biofuel potential in comparison with *Pennisetum purpureum* by Jank *et al*. ^35^. They found that even though the total yield of *P. maximum* lesser than *P. purpureum*, but *P. maximum* recorded higher leaf percentage, leaf cellulose content (29.5%) and stem cellulose (35.6%) content proving it as a better candidate for biofuel applications. Similarly, Lima *et al*.^36^ studied bioethanol potential of Brazilian grasses including *P. maximum* and reported higher cellulose (39.87%) and hemicellulose (26.62%) which makes this plant a suitable feedstock for biofuel with bioethanol potential of 285.70 L/dry ton. Kim *et al*.^37^, recorded a 54.1% increase in biomass of *Panicum varigatum* a bioenergy grass when they grow it in association with *Burkholderia phytofirmans* strain PsJN an endophytic/rhizospheric bacteria which was known to produce ACCd^38^.

The above studies were performed under normal conditions without any induced stress. Upon exposure to the stress, plants show a reduction in their growth as indicated by shoot, root length, dry matter, fresh weight and leaf area. Further, the secondary cell wall of the plants is strengthened by the incorporation of lignin and hemicellulose to avoid cell wall damage^10^. These observations were supported by increased activity of Phenylalanine ammonia lyase, a key enzyme in lignin synthesis pathway^39^. If bioethanol production is performed using such biomass, their digestibility becomes more difficult^9,40^ which increase the cost of pre-treatment and reduces the ethanol yield. Similarly, in our studies, control plants exposed drought and salt stress yielded less total reducing sugars in comparison with rhizobacteria treated and normal plants. Subsequently, the recalcitrance of cell wall for pretreatment and enzymatic hydrolysis leads to decreased ethanol yield.

In agreement with above reports, our results revealed that control plant (not treated with bacteria) exposed to salt and drought stress recorded a significant (P ≤ 0.05) decrease in shoot and root length, dry weight in comparison with the normal plant. Under similar stress conditions, ACCd bacteria treated seedlings recorded significant improvement in plant growth which was near equivalent to plants grown under normal conditions. Similar observations were reported by Li *et al*.^41^ and Gagne-Boarque *et al*.^42^, where applications of beneficial endophytic/rhizobacteria improved the growth of Elephant grass (*Pennisetum purpureum* Schumach) and model grass (*Brachypodium distachyon*) under salt and drought stress. Further cell wall composition analysis of control plants concerning normal plants revealed a significant (P ≤ 0.05) decrease in cellulose and thickening in secondary cell wall due to increased deposition of lignin. These observations are in agreement with earlier reports^10,39,40^. The plants treated with ACCd producing rhizobacteria recorded tendency to maintain the cell wall composition closer to normal plants (Fig. 3a,b). All the test bacteria recorded an increase in cellulose, hemicellulose and a decrease in lignin content concerning control seedlings.

## Methods

### Biological samples

A field survey was conducted to collect rhizospheric soil, during January- April 2014 in the regions of Jhansi (UP), India. Organically grown *P. maximum* field identified, and rhizospheric soils were collected from the five randomly selected plants from each plot. These samples were pooled to get a composite sample and transported to the lab for further analysis within 24 h.

Seeds of *P. maximum* (Jacq.) cultivar BG-2 was procured from seed stock maintained at Indian Grassland and Fodder Research Institute (IGFRI), Jhansi, Uttar Pradesh, India. The seeds were surface sterilized with 1% sodium hypochlorite for 1 min followed by three-time washing with tap water, blot-dried and used through the experiment.

### Isolation of ACC utilizing rhizobacteria

Loosely adhered soil samples on root surface were removed by gentle shaking. Roots were cut into 1 cm bits with the adhered soil samples using a sterile blade and vigorously stirred with PBS containing Tween 20 for 15 min. This homogenized mixture was filtered through four layers of muslin cloth, and the filtrate served as stock for the isolation of ACCd bacteria. The stock solution was serially diluted and 100 μl of each dilution was spread plated on DF salt minimal media containing ACC as sole N source^43^. The plates were incubated for 48 h at 30 ± 1 °C and bacteria grown were pure cultured onto Nutrient Agar (NA).

Quantification of ACC utilizing ability of isolated rhizobacteria was done as explained by Li *et al*.^44^. A standard curve was prepared using a different concentration of ACC (0.001 to 1 mmol/L) and used to quantify the ACC present in the medium after incubation with bacteria. ACC deaminase activity was determined by following the method of Penrose and Glick^43^ which measures the quantity of α-ketobutyrate and ammonia released due to the cleavage of ACC by the activity of ACC deaminase. The enzyme activity was expressed in nmol of α-ketobutyrate mg/protein/h.

### Identification of rhizobacteria

Gram’s nature, morphology and endospore were determined following the standard procedures. The bacteria were subjected to various biochemical tests and results were analyzed^45^. Further, the identity of bacteria was confirmed by amplifying and sequencing 16 s rRNA gene. Briefly, bacteria were grown in nutrient broth (NB) for 24 h and pelleted by centrifugation. The bacterial pellet was washed with Phosphate buffer saline (PBS) three times and used for DNA extraction. Bacterial DNA was isolated using Bacterial DNA purification kit (Himedia, India) following the manufacturers’ instruction. Forward primer (8F) 5′-AGAGTTTGATCCTGGCTCAG-3′ and Reverse primer (1492 R) 5′-GGTTACCTTGTTACGACTT-3′ were used for the amplification of 16 s rRNA gene. After the PCR reaction amplified product was visualized on 1% agarose, purified and sequenced. 16 S rRNA gene sequence was aligned with the reference sequence already published in the NCBI database using BLAST algorithm by following the method of Altschul *et al*.^46^. The 16 s rRNA gene sequences were deposited in the NCBI database and accession numbers were obtained.

### Characterization of rhizobacteria for beneficial traits

The ACCd rhizobacterial isolates were analyzed for their various beneficial traits such as root colonization, phosphate solubilization, indole acetic acid production, biofilm formation, siderophore production, hydrogen cyanide production and antagonistic activity following standard procedures^27,47–49^.

All ACCd rhizobacteria were examined for their potential to tolerate salt and drought following microtiter plate method. For water stress, each well of the microtiter plate was filled with sterilized 250 μl Nutrient broth (NB) amended with different concentration of PEG (MW 6000) ranging from 0–30% corresponding to final osmotic potentials 0 to −0.9 M pa, respectively^50^. Ten μl bacteria suspension (OD 0.45 at 610 nm) was inoculation each well. In the case of salt stress, different concentration of NaCl ranging from 10 to 250 g/L was incorporated to NB, followed by inoculation with 10 μl test bacteria. The inoculated plates were incubated at 35 ± 1 °C for 24 h on a rotary shaker at 250 rpm in a humid chamber and the bacterial growth was measured by reading at 610 nm in microtiter plate reader (Epoch). The concentration of PEG and NaCl which suppress the 50% bacterial growth was calculated and tabulated as IC_50_.

### Plant growth promotion studies

Test bacteria were grown in NB for 24 h and harvested by centrifugation (8000 rpm for 10 minutes). The bacterial pellet was washed twice with sterile saline and optical density (OD) was adjusted to 0.45 at 610 nm. Seed bacterization of *P. maximum* was done by soaking in bacterial suspension amended with 0.4% Carboxymethyl cellulose (CMC) as a binding agent. Seeds treated with distilled water amended with CMC served as control. The setup was incubated for 30 min at 30 ± 1 °C on a rotary shaker at 150 rpm. Bacterized seeds were sown thickly in pots (9 cm diameter) containing pre-sterilized potting mixture (soil: sand: Farmyard manure, 2:1:1) and maintained in a poly house with natural light and Relative humidity of 65–80%. To maintain optimal moisture level pots were watered regularly. Ten days after sowing, the seedlings were thinned to six per pot. Twenty-day old seedlings were carefully uprooted without damaging root system and soil adhered to root was removed by washing under running tap water and blot-dried. RL and SL of seedlings were immediately measured and tabulated. These plant materials were dried at 50 °C for 2–3 days (until the plant materials attain constant weight) and DW was calculated.

### Screening rhizobacteria for their efficacy to induce drought and salt stress tolerance in host plant

Bacterial inoculum preparation, seed treatment and plants were raised as explained earlier. Bacterized seeds were thickly sown and after ten days seedlings were thinned to maintain six seedlings per pot. 24 day-old-seedlings were exposed to drought stress by withholding water continuously for six days at which plants showed typical symptoms of drying. For imposing salt stress, 20-day old seedlings were watered with salt solution (100 mM) at 48 h of intervals for ten days. In both the cases towards the end of treatment period seedlings were uprooted carefully without damaging the root system and subjected to different physical, physiological and biochemical characterization. Seeds treated with distilled water amended with CMC served as a control (C) for both the experiment. For comparative analysis, a set of seedlings raised from non-bacterized seeds grown under normal conditions (N) (without drought or salt stress) was maintained throughout the experimental period. RL, SL, FW and DW of the seedlings was analyzed as explained earlier.

Relative water content (RWC), Electrolyte leakage (EL), Total chlorophyll (TC), Proline content (PC), Total soluble sugars (TSS), Soluble protein (SP), Crude protein (CP) and Total phenolic content (TPC) of control, normal and treated seedlings were determined following standard procedures.

### Chemical analysis of biomass for biofuel potential

Cellulose content in the *P. maximum* was estimated by the method of Updegraff (1969)^51^. Quantification was done by making a standard of cellulose in the range of 0 to 100 µg/ml. The hemicellulose was calculated by the difference between neutral detergent fiber (NDF) and acid detergent fiber (ADF)^52^. In case of NDF sample was refluxed with a solution made up of sodium lauryl sulfate, disodium dihydrogen EDTA, sodium borate (decahydrate), disodium hydrogen phosphate and ethoxyethanol while in ADF samples were refluxed in cetyl trimethyl ammonium bromide reagent made in 1 N H_2_SO_4_. The determination of lignin was done by the method of Goering and Van Soest^53^.

### Enzymatic Hydrolysis and Theoretical ethanol yield

Enzymatic hydrolysis of biomass (0.6 g of milled grass) was done after the pre-treatment (2% NaOH, 121 °C, and one h). The pretreated substrate was treated with five mL of 0.05 M sodium citrate buffer (pH 4.8) containing cellulase (60 U/g DM) and xylanase (1200 U/g DM). After the addition of the enzymes, the samples were incubated (50 °C, 150 rpm) for 72 h. Total reducing sugar was analyzed by following the di-nitro salicylic method (DNS) method^54^. The conversion of cellulose and hemicellulose was calculated by using the following formula,

$$B/A\times 100$$

where B is total reducing sugar after enzymatic hydrolysis and A is cellulose and hemicellulose before enzymatic hydrolysis^55^. Theoretical ethanol yield (TEY) was calculated in relation to dry matter: 0.511 g ethanol/1.0 g dry matter by considering that all glucose is available for fermentation^56^.

### Experimental design and Data analysis

All beneficial traits of ACCd bacteria were analyzed in triplicates and repeated thrice and the average value is represented. Under poly-house conditions, each treatment (normal, control, salt, drought and bacterial) contained three sets of eight pots each and were arranged in randomized order. Poly house experiments were repeated thrice and the value obtained were averaged and tabulated.

All data obtained from laboratory and poly house experiments were statistically analyzed through analysis of variance (ANOVA) using *SPSS* Windows (version *16.0*). Probabilities of significant difference from ANOVA were used to test the significance among treatments (P ≤ 0.05).

## Conclusion

By analyzing the results, it could be concluded that ACCd rhizobacteria modify the stress response of host-plant, which lead to the improvement of biomass characters. The method of assigning weight to beneficial traits to select best bacteria was found more suitable and convenient to screen bacteria in large numbers. Bacterial treated plants exposed to stress showed a tendency to maintain the growth parameters and cell wall composition closer to normal plants. The current study provides an opportunity to understand rhizobacteria-host interaction under abiotic stress and apply the same for the cultivation of fuel crops on the marginal and degraded land.

## Electronic supplementary material

Table S1, Table S2, Figure S1, Figure S2.
