# Supplementary material for: 1-Aminocyclopropane-1-carboxylic acid deaminase producing beneficial rhizobacteria ameliorate the biomass characters of Panicum maximum Jacq. by mitigating drought and salt stress
Source: Sci Rep. 2018 Nov 30;8:17513. doi: 10.1038/s41598-018-35565-3 (PMC6269535; doi:10.1038/s41598-018-35565-3)
Supplement: Supplementary file 1 — Table S1, Table S2, Figure S1, Figure S2. [file 41598_2018_35565_MOESM1_ESM.pdf]

Supplementary information

to

1-Aminocyclopropane-1-carboxylic acid deaminase producing beneficial rhizobacteria ameliorate the biomass characters of *Panicum maximum* Jacq. by mitigating drought and salt stress

Garima Tiwari[1] , Duraivadivel P[2], Satyawati Sharma[1], and Hariprasad. P.[2,*]

Affiliation

[1]Biochemistry Lab, Centre for Rural Development and Technology, Indian Institute of Technology Delhi, Hauz Khas, New Delhi 110016.

[2]Environmental Biotechnology Lab, Centre for Rural Development and Technology, Indian Institute of Technology Delhi, Hauz Khas, New Delhi 110016.

Table S1. ACC-utilizing bacteria tested in the present study

S.No	Isolates	Closest matching species	Accession Number
1	5JB	*Bacillus licheniformis*	KY449413
2	20B	*Bacillus licheniformis*	KY468996
3	7C	*Bacillus species*	NS
4	11-2E	*Pseudomonas species*	NS
5	22F2	*Bacillus licheniformis*	KY468997
6	22F1	*Bacillus licheniformis*	KY468998
7	4F1	*Bacillus licheniformis*	KY468999
8	3B	*Bacillus licheniformis*	KY469000
9	11-2F	*Uncultured citrobacter*	NS
10	20N	*Bacillus licheniformis*	KY469001
11	8F	*Pseudomonas species*	NS
12	11G	*Bacillus licheniformis*	KY469002
13	2L	*Uncultured citrobacter*	NS
14	14P	*Alcaligensfeacalis*	KY469003
15	3E	*Paenibacillus alvei*	KY469004
16	4F11	*Pseudomonas species*	NS
17	18D	*Bacillus licheniformis*	KY469005
18	14N	*Bacillus licheniformis*	KY469006
19	3C	*Bacillus licheniformis*	KY469007
20	5C	*Bacillus licheniformis*	KY469008
21	20F	*Pseudomonas species*	NS
22	23*	*Pseudomonas species*	NS
23	18G	*Bacillus licheniformis*	KY558747
24	4F2	*Pseudomonas species*	NS
25	23G	*Bacillus subtilis*	KY469009
26	1JF	*Bacillus licheniformis*	KY469010
27	7JG	*Bacillus subtilis*	KY558778
28	7D	*Bacillus licheniformis*	KY469011
29	24F	*Bacillus subtilis*	KY560576
30	22A	*Bacillus subtilis*	NS
31	5JE	*Bacillus subtilis*	KY561335
32	20H	*Bacillus licheniformis*	KY469012
33	14G	*Bacillus subtilis*	NS
34	11-2I	*Bacillus licheniformis*	KY469013
35	18F	*Bacillus subtilis*	KY561346
36	5JD	*Bacillus subtilis*	KY561342
37	4A*	*Bacillus licheniformis*	KY469014

NS: Not submitted.

Table S2. Physicochemical chemical characteristics of soil used for the experiments.

Physicochemical characteristics	Values obtained
Texture	Sandy Loam
EC	630 µS m-1
pH	7.46
Organic carbon	1.73%
Total nitrogen	0.51%
Available phosphorus	11.3 mg/kg
Potassium	54.3 mg/kg

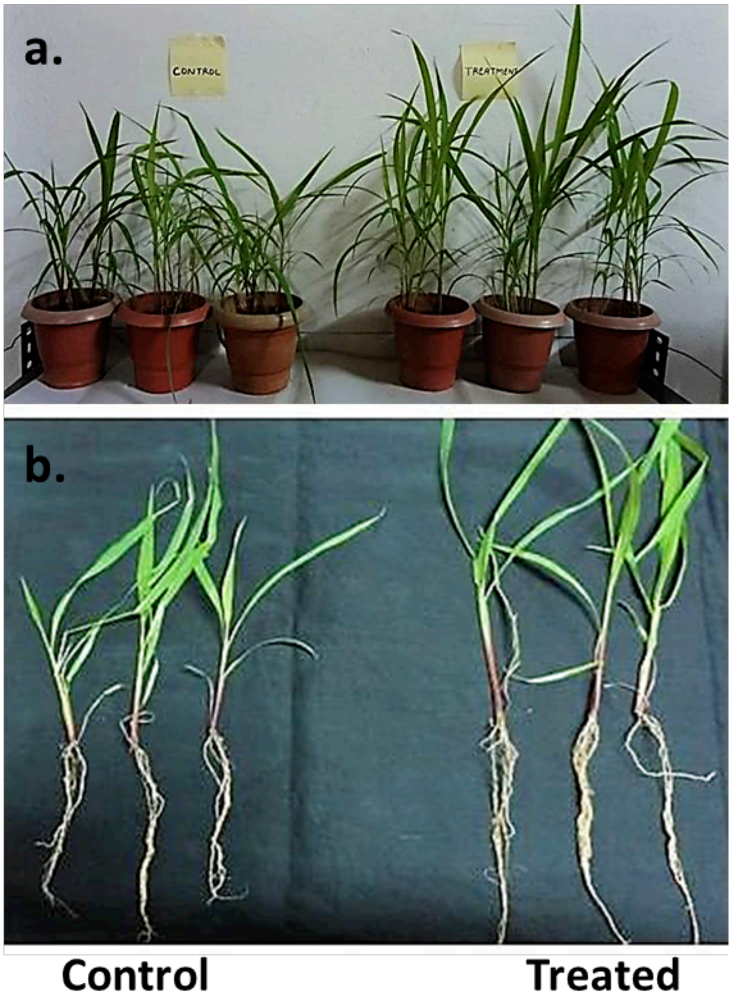

Fig S1. Plant growth promotion studies under normal condition. (a) pot experiments and (b) root and shoot length analysis.

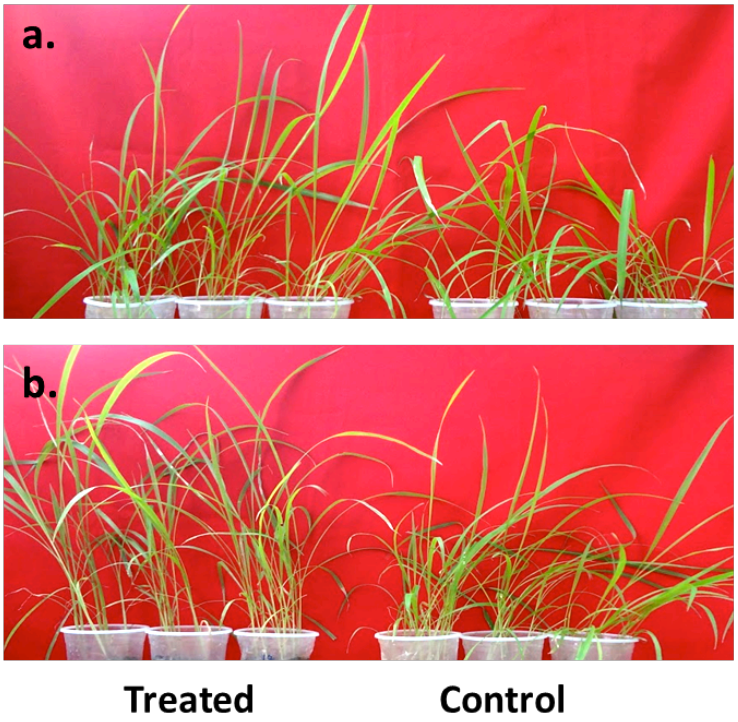

Fig S2. Control and bacterial treated plants exposed to (a) Salt and (b) Drought stress.
